# Perceptions of women enrolled in a cardiovascular disease screening and prevention in HIV study

**DOI:** 10.4102/safp.v65i1.5554

**Published:** 2023-04-24

**Authors:** Sherika Hanley, Galaletsang J. Ndlazi, Stacy T. Maddocks, Verusia Chetty

**Affiliations:** 1 Umlazi Clinical Research Site, Centre of the AIDS Research Programme in South Africa, Durban, South Africa; 2 Department of Family Medicine, Faculty of Health Sciences, University of KwaZulu-Natal, Durban, South Africa; 3 Department of Physiotherapy, School of Health Sciences, University of KwaZulu-Natal, Durban, South Africa; 4 Department of Physical Therapy, University of British Columbia, Vancouver, Canada

**Keywords:** cardiovascular disease screening and prevention, women with HIV, WHO-PEN, body image, South Africa

## Abstract

**Background:**

The ISCHeMiA (integration of cardiovascular disease screening and prevention in the human immunodeficiency virus [HIV] management plan for women of reproductive age) study is an ongoing, 3-year, prospective, quasi-experimental study comparing usual care to a primary health care intervention plan guided by the World Health Organization Package of Essential Non-Communicable (WHO-PEN) disease interventions. Sixty eight percent of women were overweight or obese at baseline in the ISCHeMiA study, many of whom reported nonadherence to interventions at 6 months post enrolment. This study explores the perceptions of women living with HIV (WHIV) towards their participation in the ISCHeMiA study to understand the barriers and facilitators to lifestyle modification interventions for cardiovascular disease (CVD) risk prevention.

**Methods:**

A qualitative enquiry using semistructured interviews was conducted with 30 overweight WHIV at one year post-enrolment in the WHO-PEN intervention arm of the ISCHeMiA study. Data were transcribed verbatim following the interviews and analysed using conventional content analysis.

**Results:**

Four major themes emerged from the data, namely perceived body image, benefits barriers and recommendations to improve adherence to WHO-PEN lifestyle modification management.

**Conclusion:**

Women in the ISCHeMiA study believed that HIV associated stigma hindered access to care. Financial limitations and the lack of social support posed barriers to adherence to programme participation. They were further challenged by poor body image perception. Participants believed that such interventions offered them hope and feelings of improved well-being. Women recommended that lifestyle modification interventions such as those studied in the ISCHeMiA study should include partners and family to improve adherence through social support.

**Contribution:**

Women living with HIV believed that lifestyle modification interventions improved their sense of wellbeing. However, HIV stigmatisation, lack of social support, and poor body image perception posed barriers to adherence to lifestyle interventions. Recommendations to improve adherence to lifestyle modification strategies include person-centred, integrated chronic disease models of care.

## Introduction

There were approximately 38 million people across the globe living with human immunodeficiency virus (HIV) in 2020.^1^ South Africa (SA) has the highest number of people living with HIV in the world,^2^ with 8.2 million of its population living with the virus, and an estimated 24% of the population are women aged between 15 and 49 years.^3^ Furthermore, women and girls make up 63% of new HIV infections in sub-Saharan Africa (SSA).^1^ Large-scale efforts have led to greatly improved access to antiretrovirals therapy (ART), with 28.0 million people accessing ART worldwide at the end of June 2021.^1^ South Africa is home to the largest ART rollout programme in the world, with over 5 million people receiving ART.^4^

Among people living with HIV, the increased prevalence of noncommunicable disease (NCD) reflects a combination of factors, including traditional NCD risk factors, direct consequences of HIV infection and exposure to specific antiretrovirals.^5^ Recent literature has highlighted the interconnectedness between HIV infection, ART and cardiovascular disease (CVD).^6^ Therefore, HIV remains a risk factor and a significant contributor to CVD-related morbidity and mortality.^7^ In women living with HIV in low- and middle-income countries (LMICs) such as SSA, cardiometabolic disease is on the rise.^8^ Cardiovascular disease has been shown to be a leading cause of death, both globally and in SA, with the related mortality surpassing that associated with infectious diseases.^9^

Traditional risk factors for CVD, such as sedentary lifestyle and unhealthy diet, appear to be more evident in people living with HIV in LMICs because of an epidemiological transition.^10^ Antiretroviral drugs can be associated with harmful changes, particularly metabolic and physical changes such as weight gain.^11^ Local data provides evidence that obesity is more pronounced in black women compared to men.^12^ Furthermore, SA is facing an epidemic of hypertension, the most dominant CVD risk factor worldwide.^8^ Recognising that the risk of developing CVD is substantially increased in people living with HIV, the World Health Organization (WHO) has made recommendations for the provision of a CVD risk assessment for all people living with HIV based on the standard protocols.^13^

The WHO developed a Package of Essential Non-Communicable (PEN) disease interventions for low resource primary care settings.^14^ The World Health Organization Package of Essential Non-Communicable (WHO-PEN) disease interventions is a set of cost-effective interventions, applicable to both population-wide and individual levels, including health education, promotion of healthy behaviours and early diagnosis of NCDs and their risk factors. It employs inexpensive technologies; affordable medications for prevention and treatment of CVD, stroke, diabetes, hypertension, cancer and asthma; regular follow-up; and referral. These low-technology interventions, if effectively delivered, can reap future savings in terms of reduced medical costs, improved quality of life and productivity.^14^

The WHO-PEN incorporates the WHO International Society of Hypertension (WHO/ISH) screening and intervention through lifestyle modification and prompt treatment of identifiable CVD risk factors in an integrated model of HIV care. The WHO-PEN implementation tools enable early detection and management of CVD to prevent life-threatening complications in people living with HIV.^14^ Studies assessing the WHO-PEN intervention in the general population have shown that it is a feasible management plan in reducing CVD risk.^15^

The integration of cardiovascular disease screening and prevention in the HIV management plan for women of reproductive age (ISCHeMiA) study is a prospective, quasi-experimental study comparing usual care with a primary healthcare intervention plan guided by the WHO-PEN.^16^ Baseline data from ISCHeMiA revealed that 68.0% of South African women of African descent had an increased body mass index (median BMI = 27 kg/m^2^).^16^ Other local studies demonstrate an increase in obesity from 34.4% to 47.0% in the first three years after HIV acquisition and have shown that obesity is more pronounced in black women compared to men.^17^

Although obesity in women is a well-known concern, and strategies have been developed for the prevention and control of obesity in SA,^18^ it will be beneficial to explore the perceptions of women living with HIV about their body image to tailor existing obesity and CVD intervention strategies. Seventy-five percent of overweight women reported being satisfied with their body image at enrolment into ISCHEMiA.^16^ At the 6-months postenrolment follow-up visits in the ISCHeMiA study, it was noted that many women were not adhering to the interventions implemented at enrolment in the study. In SA, cultural beliefs are strongly shaped by gaining weight that symbolises wealth and happiness to cover the stigma of HIV, particularly among black women.^19^

This study explores the perceptions of women living with HIV towards their participation in the WHO-PEN intervention arm of the CVD screening and primary prevention ISCHeMiA study. Participant recommendations and possible reasons for nonadherence to the interventions will assist in streamlining intervention strategies, particularly in women who may be hindered by culture-specific perceptions of body image.

## Methods

The ISCheMiA study is a CVD risk prevention and screening study nested in the multinational President’s Emergency Plan for AIDS Relief (PEPFAR) Promoting Maternal and Infant Survival Everywhere (PROMise) Ongoing Treatment Evaluation (PROMOTE) study, which aimed to evaluate long-term safety profiles in women living with HIV.^20^ The ISCHeMiA study was conducted at the Centre for the AIDS Programme of Research in South Africa (CAPRISA) Umlazi clinical research site, located in the Umlazi township on the south coast of KwaZulu-Natal from November 2018 to February 2022. All women enrolled in the WHO-PEN intervention arm were screened annually for hypertension, diabetes, high cholesterol and obesity. Participants received a lifestyle modification advice sheet at study entry which addressed recommendations for dietary intake, physical activity, smoking cessation and reduction in alcohol intake. Women who were overweight were referred for a formal group class held by the local hospital dietitian.

A qualitative design was employed to explore the perceptions and opinions of overweight women living with HIV about their body image and their experiences during their participation in the ISCHeMiA study. Purposive sampling was used to recruit 30 overweight women living with HIV on ART who had completed one year follow-up of the ISCHeMiA study.^21^ The women were telephonically invited to participate in in-depth semistructured interviews at the study site.

The researcher was trained in conducting interviews for qualitative research purposes. Interviews were held in a private room and lasted between 25 min and 35 min. Interviews continued until no new data emerged. A semistructured questionnaire was used, and interviews were audio-recorded using a Dictaphone. The researcher was impartial throughout the research process by not portraying any form of discrimination. The questions in the semistructured interview guide were as follows:

- How would you describe your experience in participating in the study (feelings, behaviour)?
- What influenced your adherence to the management offered on the programme (finance, stigma, relationships)?
- Explain how you feel about yourself (personal development, body image, self-worth).
- Do you have any recommendations to improve adherence to management (education, access issues, personal reasons)? (Appendix 1)

Data were transcribed verbatim after the interview. The isiZulu transcripts were translated into English and back-translated by a language expert to ensure rigour. Field notes were used to record the data collection process. In order to identify the common patterns and threads that featured throughout the transcript, thematic analysis was adopted to understand and interpret the feelings and behaviour of the participants.^22^ The transcribed data was read and reread independently by two researchers, firstly for the purpose of familiarisation, to obtain a general impression of the overall data, and subsequently to identify codes and emergent themes through an inductive process. Final themes were discussed with a third researcher, and this resulted in consensus regarding the four over-arching themes.

### Ethical considerations

The University of KwaZulu-Natal Biomedical Ethics Committee issued ethical approval (ref. no. BFC 220/18). A consent form and information sheet describing the purpose of the study were issued to each participant. All the women were informed about their rights to discontinue participation in the study at any point without any discrimination or compromise of care at the healthcare facility.

## Results

Thirty women were interviewed in this study during November 2019. All participants were women living with HIV and overweight or obese at the time of the study. All women were receiving efavirenz-based ART at the time of the interviews, which occurred prior to the inclusion of dolutegravir in South African HIV treatment guidelines. All the women were South African, except one who was Malawian. Table 1 depicts participants’ demographic characteristics.

**TABLE 1:** Demographic information of participants.

Characteristics	Number
**Age (years)**	
20–30	07
31–35	11
36–40	07
41–45	03
46+	02
Total	30
**Body mass index (kg/m^2^)**	
25 to < 30	03
30 to < 5	12
35 to < 40	12
≥ 40	03
Total	30
**Marital status**	
Married	05
Unmarried	24
Divorced	01
Total	30
**Language**	
Zulu	29
English	01
Total	30
**Level of education**	
No education	01
Grade R–6	00
Grade 7–10	07
Grade 11	11
Grade 12	11
Tertiary level	00
Total	30
**Level of employment**	
Employed part-time	08
Employed full-time	07
Unemployed	15
Total	30
**Medical history**	
Asthma	02
Hypertension	04
Diabetes	01
Previous TB	01
Total	08

TB, tuberculosis.

Thematic analysis of the data revealed four main themes, namely benefits of the WHO-PEN lifestyle modification intervention, barriers to the WHO-PEN lifestyle modification intervention, perceived body image and recommendations to improve adherence to the WHO-PEN lifestyle modification management. Table 2 reflects the themes as well as the subthemes, with illustrative quotes. Pseudonyms with descriptors have been used for participants to ensure confidentiality and privacy of participants.

**TABLE 2:** Themes, subthemes and quotes from women enrolled in the integration of cardiovascular disease screening and prevention in the HIV management plan for women of reproductive age study.

Themes	Subthemes	Quotes
Benefits to the WHO-PEN lifestyle modification intervention	Improved self-worth	‘Study gave me strength to face my partner and tell him that I tested HIV positive and also feel free to talk to nurse because [*she or he*] will advise me on other things.’ (Favourite, 47 years, employed, single)
		‘Was taught how to look after myself and behaviour, because having HIV is not the end of the world.’ (Xolile, 40 years, unemployed, single)
		‘I told myself that I don’t care what people say about my status, because nowadays we’re the same.’ (Promise, 31 years, part-time employment, single)
	Feelings of hopefulness	‘Being part of the study made me feel good about myself because they take care of me, compared to other people with the same status in another clinics.’ (Thabisile, 35 years, unemployed, single)
		‘In 2013 I was pregnant, and when I went for my every month clinic visit after the routine that they do, I was told that I am HIV positive and was also told that if I am interested in joining the study I can join, and since I joined the study I am 100% happy because they do things that other clinics don’t do.’ (Nompumelelo, 36 years, unemployed, single)
	Improved well-being	‘My experience in this study is that you have to look after your body now that I know I am HIV positive.’ (Thabisile, 35 years, unemployed, single)
		‘I have decided to do my own exercise at home, and sometimes I go to community gym and exercise with other members of the community.’ (Phindile, 32 years, unemployed, single)
		‘I didn’t care about taking treatment on time, but since I joined this study, I became responsible and take it in time because they told me to improve my memory so that I don’t forget now and then, because it will reduce my viral load.’ (Promise, 31 years, unemployed, single)
	Knowledge advancement	‘I have learned that I must always check out for other diseases because they care for us a lot and make us feel special amongst other people (referring to research team).’ (Slindile, 32 years, employed, single)
		‘They teach us about other diseases that are associated with HIV.’ (Busisiwe, 36 years, part-time employment, single)
		‘I was taught not to cook with cooking oil, remove skin when cooking chicken, eat healthy food and always exercise.’ (Bella, 40, unemployed, single)
Barriers to the WHO-PEN lifestyle modification intervention	Environmental barriers	‘I didn’t go to dietitians because I only get day off over the weekend, and unfortunately, they don’t work over the weekend. I work as a nanny and the child I look after, the parents work Monday to Friday.’ (Ayanda, 33 years, part-time employment, single)
		‘I don’t have any personal time for myself to lose the weight, but if I had time, I was going to create my own exercises.’ (Thabisile, 35 years, unemployed, single)
	Financial impediments	‘I sometimes face serious problem with money since I am using a taxi and I am not working. My husband is working and I also receive social grant for my children, but still it is not enough for things that needs money.’ (Zaziphi, 26 years, unemployed, married)
		‘I didn’t go to the dietitian because I didn’t have money for transport; we only survive with social grant money at home.’ (Ntombifikile, 38 years, unemployed, single)
		‘I am not working; I rely on social grant. I was told by my friend that if you go to the dietitians, you will be told to go and buy specific things to eat, so I didn’t go because I don’t have money to buy all those things.’ (Priscilla, 34 years, unemployed, single)
	Challenges of nondisclosure	‘My partner is supporting me; I even use him as my next of kin, but he is afraid of checking while he knows the importance of knowing your status.’ (Nompumelelo, 36 years, unemployed, single)
		‘My partner doesn’t know my status and I don’t know his; I tried to encourage him to test but I failed, so I did not tell him why I am participating in the study.’ (Promise, 31 years, part-time employment, single)
	Lack of social support	‘I was given a diet plan, but I can’t follow because at home they said boiled food is boring, and when I cook two pots, they say I am wasting the grocery.’ (Nomnotho, 48 years, employed, single)
		‘The father of my child, as soon as he heard that I am HIV positive, he separated from me.’ (Busisiwe, 36 years, part-time employment, single)
	Stigma experienced by women	‘I sometimes feel bad when people speak about me behind my back about my status. One of my sister-in-laws saw me at the clinic in the queue of collecting ARVs and told my son, whom I did not tell because I was waiting for the right time, and when my son told me I was hurt but accepted it later and move on. She also told my neighbours.’ (Zaziphi, 26 years, unemployed, married)
		‘I don’t like it when people see me collecting my treatment because my status is my secret, so I don’t understand why I have to collect my treatment from local clinic, because this place is not very private.’ (Nompumelelo, 36 years, unemployed, single)
Perceived body image	Poor body image	‘I can see I am gaining weight and I am not happy.’ (Busisiwe, 36 years, part-time employment, single)
		‘I have big tummy and big thighs and I am not doing anything about it; I don’t really like my body image.’ (Priscilla, 34 years, unemployed, single)
		‘My body is not looking good and I am not happy about it [*bows head sadly*]. I wish I can lose a bit of kilos.’ (Thabisile, 35 years, unemployed, single)
		‘I can see that I am gaining weight and I feel okay, even though sometimes I am not happy about it; the main problem is my stomach is too big.’ (Nomnotho, 48 years, employed, single)
		‘I didn’t know that I gain weight until I was told; I was even told things that can put me at risk of other diseases [*looks sadly at interviewer*].’ (Xolile, 40 years, unemployed, single)
	Perceived body changes associated with healthcare	‘I have increased appetite, which I think is caused by prevention injection.’ (Nomnotho, 48 years, employed, single)
		‘I feel my body image is okay; it is only the abdomen that is too big; maybe is because I had a baby with caesarean section and I did not tie my abdomen after birth.’ (Nompumelelo, 36 years, unemployed, single)
		‘My abdomen is too big; that is why I look fat. Maybe it’s because of prevention injection I use to use that I left 2 years back. I also heard that ARVs make you gain weight; maybe they are contributing to my weight gain.’ (Precious, 30 years, unemployed, single)
		‘I am not happy about my abdomen; it is too big due to the operation I underwent during childbirth.’ (Nompumelelo, 36 years, unemployed, single)
	Denialism of physical changes	‘I am actually shocked; I thought that I am losing weight but didn’t know that I am gaining. I have no words to express my feelings [*looks sadly at the interviewer*].’ (Slindile, 32 years, employed, single)
		‘I bought a bathroom scale and weigh myself now and then, and I walk a very long distance when I go to church because I think that will help me to lose weight. I am surprised that I gained weight; I was not aware.’ (Xolile, 40 years, unemployed, single)
		‘I wish I can go to the dietitians so that I don’t gain more weight, because I didn’t go when I was given a letter. There is nothing wrong with my body image. If I was meant to lose weight, I was going to lose because I walk long distance to go to work, which is an exercise to me.’ (Nompumelelo, 36 years, unemployed, single)
		‘I was told the importance of a healthy diet and exercise in my life, but I never follow because I was not worried about gaining weight. I didn’t even follow the diet plan that was given.’ (Mathombi, 38, unemployed, single)
Recommendations to improve adherence to the WHO-PEN lifestyle modification management	Improve waiting times	‘We can complain about time because we are people, but to be realistic, things that they do to us and check really take time, so for us is just to be patient.’ (Ntombizodwa, 33 years, employed, single)
		‘If they can improve in time and speedy the process it will be better, because we sit here the whole day and end up getting tired, but at least they give us something to eat while waiting.’ (Nomathemba, 28 years, unemployed, single)
	Improve social support	‘Please include our partner in the study so that all the information we get here, they can also get.’ (Xolile, 40 years, unemployed, single)
		‘We will appreciate if our family can also be part of the study so that we can speak the same language at home, especially when it comes to following the diet and cooking in one pot the healthy food.’ (Nomnotho, 48 years, employed, single)
		‘It will make me feel OK and strong if my family and boyfriend were also learning in clinic, now I feel scared of other people where I live [*referring to community and family*].’ (Nomathemba, 28 years, unemployed, single)

HIV, human immunodeficiency virus; ARV, antiretrovirals; WHO-PEN, World Health Organization Package of Essential Non-Communicable.

The theme ‘benefits to the WHO-PEN lifestyle modification intervention’ describes the benefits that the women experienced while participating in the ISCHeMiA study. There were reports by participants who felt an improved self-worth that gave them the courage to disclose their status to their partner and family, as well as feelings of hopefulness following their enrolment in the ISCHeMiA study. Some women believed that their general well-being improved following their engagement in the ISCHeMiA study, while others felt that their knowledge about their health was enhanced following participation in the study.

The theme ‘barriers to the WHO-PEN lifestyle modification intervention’ highlighted the barriers experienced by women in the study. Some participants felt that the environmental barriers such as travel to sites and financial limitations hindered their participation in the study. Furthermore, there were women who mentioned work commitments and time constraints as reasons for them being unable to access care offered by the programme during the week. Some of the women felt that barriers to participating in the interventions offered in the ISCHeMiA programme included nondisclosure of their HIV status to their family and/or partners, which made it difficult to attend the appointments. A lack of social support from their family and partners hindered their progress. Women felt burdened by stigma, and this posed a challenge to them accessing care.

The theme ‘perceived body image’ encompassed the perceptions of women of themselves. Some women felt they were gaining weight, and a few women believed that their ART and contraception were contributing to their weight gain. There were some participants who denied the changes in their body. Women expressed that their body image and changes they experienced in their body composition were leading to feelings of unhappiness.

Participants in the ISCHeMiA study recommended that in order to improve adherence to the WHO-PEN lifestyle modification and attendance to the appointment visits, organisational changes such as reducing waiting times need to be addressed. Moreover, women recommended that it would help adherence to programmes such as the ISCHeMiA study if family and partners were included in the care approach to offer support to participating women.

## Discussion

This study explored the perceptions of women living with HIV towards their participation in the ISCHeMiA study. Feelings of hopefulness were described by most women as a benefit to participation in the ISCHeMiA study. They found the treatment approach at the study clinic site to be thorough and holistic and had a general sense that their physical and psychological needs were met.

Participants positively viewed their participation in the ISCHeMiA study as an opportunity for knowledge advancement with regard to their health and well-being. They expressed appreciation for the information that they received and felt they understood more about their health and medical approach associated with HIV and other diseases, including leading a healthy lifestyle. Although they enjoyed the advice and approach to care that they received, women expressed challenges with adhering to their clinic appointments because of resource constraints.

Women felt that participation in the ISCHeMiA study afforded them support and helped them with their HIV status disclosure to their social networks. Women described feeling less stressed, and they were more willing to be responsible with the care of their bodies and the adherence to treatment because of positive relationships between them and their healthcare providers. Strong patient–healthcare provider relationships are essential for the improvement of engagement in care.^23^ In a study by Clouse et al. which explored the utilisation of healthcare among post-partum women living with HIV, they found that increased HIV status disclosure to loved ones improved participants’ levels of self-care as well as adherence to clinic appointments.^24^ Similarly, most of the participants gained confidence to disclose their HIV status to their loved ones and reported feelings of improved self-worth. Positive disclosure beliefs have been associated with greater self-esteem and an increased level of status disclosure and quality of life.^25^

Maman et al. highlighted that patients tend only to disclose their status to those who they believe would support them.^26^ This study’s participants believed that once they received support from family members who were aware of their status, they would be confident to face the community and adhere to lifestyle modification programmes such as those offered in the ISCHeMiA study.

Nondisclosure of HIV status was an inhibitor to participation in the WHO-PEN intervention for some of the participants. Attempts were made by some women to convince their partners to test for HIV as a couple, as they believed that this would help them overcome their fear of disclosure, but their attempts were met with refusal from their partners. These findings are in keeping with the existing literature in which there is a reduced influence by women on their partners to test for HIV.^27,28^ Additionally, women believed that stigmatisation limited their access to care through the programme. They were embarrassed to wait in the queue at the clinic and be seen by community members. These experiences are common with HIV access programmes, including HIV testing and disclosure.^29^ Yehia et al.^23^ found that retention in HIV care programmes was largely affected by the fear of stigma, and this phenomenon has been well reported in other studies.^30,31^ In order to improve access to care, misperceptions around HIV need to be addressed in poor resourced communities such as SA, through large community-based education initiatives.^32^ Moreover, the ongoing transition to integrated models of chronic care will aid in the reduction of stigmatisation by moving away from segregated HIV clinics.^33^

Although all interviewed participants were overweight or obese, some of the women did not associate their weight with poor body image perception and were mostly accepting of their physique. A few women associated their weight gain with HIV treatment and contraception usage, many of which are well-established risk factors for weight gain in women.^34^ Consideration of ART type, hormonal status and pregnancy history has the potential to improve prevention and outcomes of CVD in women.^35^ A study by Devanathan et al. described positive connotations of being overweight rather than underweight among black South African women with other pre-existing NCDs.^36^ Among black South Africans, being slim is often associated with illnesses, including HIV.^37^ These perceptions could possibly lead to barriers to lifestyle adherence modification advice around dietary and exercise recommendations. Such perceptions need to be addressed via culturally sensitive patient–healthcare provider discussions weaved into the lifestyle modification management to promote good health and well-being.

Participants recommended that shorter waiting times at the clinic would improve adherence to lifestyle modification management approaches such as the ISCHeMiA study. Women balance many competing demands, including work (home and formal employment) and their role as caregivers. Furthermore, resource constraints impeded access to care. Resource limitations and challenges with accessing healthcare services are still too common in many public South African healthcare settings.^23,24,38,39^ Studies have evidenced that such environmental and organisational inhibitors pose a challenge to people living with HIV in resource-poor communities such as SA, emphasising the need for community outreach programmes and decentralised care.^40^ Additionally, participants believed that engaging family and partners and recruiting them as part of the programme would improve adherence to care. This recommendation is in keeping with studies which highlight the advantages of family engagement and social support to facilitate better adherence to the lifestyle modifications.^25,26,41^

Under-recognised and poorly researched CVD risk factors which disproportionately affect women include psychosocial risk factors such as depression, socio-economic deprivation, intimate partner violence and inadequate health literacy and warrant further research in settings such as SA.^42^ Additionally, research gaps need to be identified with regard to perceptions and awareness of CVD risk in women living with HIV to guide educational programme development.^43^ Primary care plans and policies in SA around caring for women living with HIV should include a holistic approach by incorporating the biopsychosocial model of healthcare that is accessible. The recommendations offered in this study provide such insight by advocating for social and psychological support as well as addressing personal barriers to improve CVD screening and prevention programme uptake and adherence in women living with HIV.

### Limitations

This study was conducted in one district of the eThekwini region and therefore represents the lived experience of women living with HIV about their participation in the ISCHeMiA study in this context. A generalisation of the results obtained from this study may not be possible, as perceptions may differ in other studies in different districts and contexts of SA. By allowing the participants to answer freely, there was a strong bias towards a discussion of HIV disclosure, weight and body image in the interview process. The authors believe that the theme of HIV disclosure emerged as a result of participant co-enrolment into the PEPFAR PROMOTE (HIV) and ISCHeMiA (CVD in women living with HIV) studies. This raises awareness for the need to integrate disease prevention and management, as HIV remains a significant barrier to access all types of care. Weight and body image were most likely influenced by 6-monthly administered ISCHeMiA study questionnaires which included specific questions related to body image perception. Furthermore, the dietary and exercise modifications were perceived to be linked directly to weight loss, which is a more tangible outcome of the intervention. Moreover, the low prevalence of hypertension, diabetes and tobacco and alcohol use in the interviewed participants led to the discussion being devoid of these risk factors.

## Conclusion

Women living with HIV believed that there were benefits to the WHO-PEN lifestyle modification interventions as incorporated into the ISCHeMiA study; however, barriers to participating and adhering to the programme do exist. Benefits of participating in the ISCHeMiA study gave women a sense of hopefulness and feelings of improved well-being. These changes in their care approach helped them to disclose their status to their social network. Participants felt a greater depth of understanding of their health and well-being. However, some of the challenges they experienced included stigma associated with HIV, and this hindered their access to care. Furthermore, their financial limitations and the lack of social support posed barriers to adherence to lifestyle modification programme participation. These were further challenged by poor body image perception.

### Recommendations and further actions

Recommendations to improve adherence and sustainability of CVD risk prevention strategies such as those accessible in the ISCHeMiA study include:

- integrated chronic disease models of care to reduce HIV-related stigmatisation
- foster encouraging and supportive relationships between patients and healthcare providers
- reduce clinic waiting times and consideration for weekend clinics to accommodate caregiver and work-related commitments
- address psychosocial aspects such as poor body image through counselling and support
- the inclusion of social networks such as partners and family members.

## Appendix 1: Interview guide

**Table:**

Demographic data sheet				
Patient name:				
Patient diagnosis:				
Comorbidities:				
		
		
Date of interview:				
Age:				
Marital status:				
Ethnicity or race:				
Level of education:				
Met inclusion criteria:			YES	NO
Participant identification number:				

**Table:**

Semistructured interview				
No.	Question	Probes
1.	How would you describe your experience in participating in the study?	Feelings Behaviour
2.	What influenced your adherence to the management offered on the programme?	Finance, time Stigma, body image Relationships
3.	Explain how you feel about yourself.	Personal development Body image Self-worth
4.	Do you have any recommendations to improve adherence to management?	Education Access issues Personal reasons
